# Cancer Biomarkers - Emerging Trends and Clinical Implications for personalized treatment

**DOI:** 10.1016/j.cell.2024.02.041

**Published:** 2024-03-28

**Authors:** Antonio Passaro, Maise Al Bakir, Emily G. Hamilton, Maximilian Diehn, Fabrice André, Sinchita Roy-Chowdhuri, Giannis Mountzios, Ignacio Witsuba, Charlie Swanton, Solange Peters

**Affiliations:** 1 Division of Thoracic Oncology, European Institute of Oncology IRCCS, Milan, Italy; 2 Cancer Evolution and Genome Instability Laboratory, The Francis Crick Institute, London, UK; 3 Cancer Research UK Lung Cancer Centre of Excellence, University College London Cancer Institute, London, UK; 4 Cancer Biology, Stanford University School of Medicine, Stanford University, Stanford, California; 5 Department of Radiation Oncology, Stanford Cancer Institute, Stanford University School of Medicine, Stanford, CA; 6 Gustave-Roussy Cancer Center, Paris Saclay University, Villejuif, France; 7 Department of Anatomic Pathology and Translational Molecular Pathology, The University of Texas MD Anderson Cancer Center, Houston, TX, USA; 8 Fourth Department of Medical Oncology and Clinical Trials Unit, Henry Dunant Hospital Center, Athens, Greece; 9 Department of Translational Molecular Pathology, The University of Texas MD Anderson Cancer Center, Houston, TX, USA; 10 Department of Oncology, University College London Hospitals, London, UK; 11 Department of Oncology, Centre Hospitalier Universitaire Vaudois (CHUV), Lausanne, Switzerland

## Abstract

The integration of cancer biomarkers into oncology has revolutionized cancer treatment, yielding remarkable advancements in cancer therapeutics and the prognosis of cancer patients. The development of personalized medicine represents a turning point and a new paradigm in cancer management, as biomarkers enable oncologists to tailor treatments based on the unique molecular profile of each patient’s tumor.

In this review, we discuss the scientific milestones of cancer biomarkers and explore future possibilities to improve the management of patients with solid tumors. This progress is primarily attributed to the biological characterization of cancers, advancements in testing methodologies, elucidation of the immune microenvironment, and the ability to profile circulating tumor fractions. Integrating these insights promises to continually advance the precision oncology field, fostering better patient outcomes.

## Introduction

A biomarker refers to any biological marker found in blood, body fluids, or tissues that signals the presence of normal or abnormal biological processes, conditions, or diseases.^1,2^ When tailored to the field of oncology, a cancer biomarker specifically identifies characteristics of cancer, ideally with a high degree of accuracy and reliability, reported as their sensitivity and specificity. The use of cancer biomarkers extends beyond merely determining the type of cancer a patient suffers from. Indeed, once a diagnosis is established, tumor markers can provide valuable insights into the likely progression of the disease, including the chances of recurrence and the expected outcomes of treatment. Cancer biomarkers play a crucial role in outlining the prognosis of a disease independently of any treatment (known as prognostic biomarkers) or in predicting how a cancer will respond to a specific treatment, which helps anticipate treatment outcomes (referred to as predictive biomarkers).

Numerous cancer biomarkers have been identified and are primarily classified according to the presence of proteins within different functional categories, such as enzymes, hormones, antigens, and receptors. Alterations in cancer-related genes, including mutations, amplifications, and translocations at the single gene level, or the creation of genetic profiles through microarrays, lead to unique genetic signatures. These changes contribute to the identification and categorization of cancer biomarkers, aiding in the understanding and treatment of the disease.^2,3^

Regardless of their type, the ideal characteristics of cancer biomarkers include methods of detection that are straightforward, reproducible, reliable, and cost-effective, all of which should correlate with demonstrable enhancements in patient outcomes. This review will explore the current applications and future prospects of cancer biomarkers, considering both the advancements in testing technologies and a more profound comprehension of tumor biology. The discussion will cover how these developments contribute to the effective use of biomarkers in cancer diagnosis, prognosis, and treatment planning.

## Biomarkers for Cancer Detection, Diagnosis and Subclassification

In recent decades, the discovery and development of cancer biomarkers have evolved through meticulous exploration of substances in tissue, but also the biological fluids of cancer patients, encompassing hormones, enzymes, and proteins.

Such markers were mainly discovered by introducing immunological techniques, like the radioimmunoassay. The rapid expansion of biological sciences has markedly driven technological advancements. Over successive decades, this progress has led to the emergence of sophisticated analytical methodologies, notably in mass spectrometry and the development of protein and DNA arrays. These methodologies are intricately linked to specific chronological eras and categorizations within the field of biomarker discovery. Genome sequencing, a pivotal advancement, has significantly enhanced the identification of oncogenes and tumor-suppressor genes. This has catalyzed the discovery of cancer biomarkers, which now serve as comprehensive tools for screening, diagnosis, prognosis, and prediction. Initially rooted in empirical observations, early cancer biomarker investigations have evolved alongside modern testing technologies. The transition from serial to parallel testing enables the simultaneous identification of multiple markers, providing insights into complex disease patterns. Present-day cancer biomarkers encompass diverse elements, including DNA, RNA, proteins, metabolites, and dynamic processes like apoptosis and angiogenesis, showcasing a rich array of attributes and combination patterns.

In the process of biomarker evaluation and development, preclinical screening employs gene-expression profiling or mass spectrometry to identify potential cancer markers. Upon discovery, a clinical assay is developed to non-invasively detect the chosen biomarker in tumor tissue or biological fluid, distinguishing positive and negative results. This assay is applied in clinical studies for screening (e.g., serum PSA in prostate cancer), diagnosis (e.g., identifying EGFR mutation in suspected lung cancer without histology confirmation), prognosis (e.g., hormone receptor status in breast cancer), and predicting treatment response (e.g., gene signatures for immunotherapy in various tumors). Despite study design biases and technical artifacts affecting single cancer biomarker history, they find applications in diagnosis (e.g., Bence-Jones protein in myeloma), prognosis (e.g., hCG in testicular cancer), and predicting treatment outcomes (e.g., ALK gene rearrangements guiding treatment in lung cancer).

The ideal cancer biomarker should possess attributes that facilitate easy, reliable, and cost-effective assessment, coupled with high sensitivity and specificity. Additionally, it should demonstrate remarkable detectability at early stages and the capacity to accurately reflect tumor burden, enabling continuous monitoring of disease evolution during treatments. However, their low diagnostic specificity is a significant limitation in the real-world applicability of some biomarkers, especially the older blood-based ones like CEA, Ca125, and Ca15-3. This limitation arises from their potential expression by non-tumor tissues, introducing a risk of misinterpretation. The implications of this limitation are multifaceted. Firstly, there is a risk of patients undergoing incorrect or unnecessary treatments due to the need for more precision in diagnosis. This poses potential health risks and raises concerns about the economic burden associated with ineffective interventions. Moreover, the low specificity of these biomarkers may result in the oversight of actual cancer cases, leading to delayed or missed detections. This delay in diagnosis could significantly impact patient outcomes and overall survival rates. Healthcare systems may be reluctant to invest in or integrate these biomarkers into routine screenings and diagnostics if there are concerns about the cost-effectiveness and overall impact on healthcare budgets. By delving into these points, a more holistic case can be built to underscore the urgency and significance of advancing oncology biomarkers. Improved biomarkers can enhance the accuracy of cancer diagnosis and treatment and contribute to economic efficiency by minimizing unnecessary interventions and optimizing healthcare resource allocation. This broader perspective aims to evoke excitement about the transformative impact of better oncology biomarkers on patient care, healthcare systems, and societal well-being.

Despite the potential drawbacks associated with single marker evaluations, such as lower specificity in prognostic or predictive roles compared to comprehensive approaches like assessing co-occurring gene alteration patterns, there are pragmatic reasons for their continued use. The simplicity and cost-effectiveness of analyzing single markers make them attractive when resources are limited or when a straightforward diagnostic or prognostic tool is sufficient. Some single markers have undergone extensive clinical validation, showcasing their reliability and accuracy in specific contexts. These well-established markers may continue to play a crucial role in routine clinical settings where their clinical utility has been firmly established. Therefore, while acknowledging the limitations, single markers’ practical advantages and validated efficacy support their continued relevance in specific molecular diagnostics and personalized medicine applications.

This applies also to immune biomarkers, which today serve as indispensable tools in cancer therapy, offering insights that guide treatment decisions and enhance the effectiveness of immunotherapies. By evaluating factors such as PD-L1 expression, tumor-infiltrating lymphocytes (TILs), and molecular signatures within the tumor microenvironment, clinicians can predict and monitor responses to immunotherapy. Additionally, biomarkers like microsatellite instability (MSI) and mismatch repair deficiency (MMR) help identify patients more likely to benefit from immune checkpoint inhibitors. The role of immune biomarkers extends beyond mere prognostication; they play a pivotal role in shaping personalized and targeted approaches, fostering a deeper understanding of the intricate interplay between the immune system and cancer cells. As our knowledge of these biomarkers continues to evolve, their integration into clinical practice promises to optimize cancer treatment strategies and improve patient outcomes. In this view, a pan-cancer approach would gain reliability regarding immune signatures able to predict the ability of tumor antigen recognition and T-cell response initiation.

In addition, the same identified biomarker might have different roles in different tumors and with different drugs (e.g., HER2 mutations, amplifications, and overexpression represent specific biomarkers for lung, breast, and gastric cancer, respectively, requiring different testing methods accordingly).

Conversely, the agnostic approach represents a paradigm shift in this scenario, which applies to two scenarios: the same biomarker across tumor types (e.g., *NTRK* gene rearrangements) and biomarker agnostic use of targeted drugs. This latter approach is at the basis of the emergence of antibody-drug conjugates (ADCs) across different tumor types. Indeed, ADCs use target antigens commonly present on tumor cells, not normal cells, to deliver cytotoxic drugs more selectively within tumor sites. As such, the presence of the target on tumor cells is theoretically required – as per historical data, but its specific quantification as a biomarker is not needed. The activity of the currently available ADCs was demonstrated regardless of the target’s expression levels across most tumors, across different targets (e.g., TROP2, HER3), and the evaluated ADCs. This critical consideration led to the repurposing of previously excluded biomarkers, as it happened for the newly identified ‘HER2 low’ breast cancer receiving benefits with the ADC trastuzumab-deruxtecan.

As a take-home message, elevating cancer biomarker research requires shifting towards multiparameter approaches, incorporating dynamic processes and immune signatures. Embracing simplicity without compromising specificity will propel the development of reliable, cost-effective biomarkers, offering transformative potential in personalized cancer diagnosis and treatment.

## Biomarkers to Inform Treatment Decision-Making: from Oncogenes to Multidimensional Assessment of Biology

Technologies such as immunohistochemistry (IHC) or polymerase chain reaction (PCR) remain standard basic tools given their affordable cost and rapidity. However, the use of next-generation sequencing (NGS) technology, enabling the rapid and cost-effective sequencing of large amounts of DNA or RNA, gradually increased, specifically for defined cancer types such as colorectal and non-small cell lung cancer and melanoma.

Recent advances in cancer research leverage advanced genetic analysis techniques, such as whole exome sequencing (WES), whole genome sequencing (WGS), and RNA sequencing (RNA Seq). WES delves into protein-coding DNA regions, shedding light on pertinent genetic changes in cancer. WGS takes a more comprehensive approach, sequencing the entire DNA, including non-coding areas, offering a holistic view of the genetic landscape. RNA Seq scrutinizes RNA molecules to decipher gene expression patterns. These methodologies collectively advance our comprehension of cancer pathways, contributing to the refinement of targeted diagnostics and therapies.

Expanding the scope, considering WES, WGS, and RNA Seq for DNA and RNA analysis, aims to deepen our understanding of oncogenic pathways. This approach becomes especially pertinent when targeted panels fail to identify actionable molecular abnormalities. The insights into molecular mechanisms have paved the way for developing drugs specifically targeting these mechanisms. Typically, these drugs prove effective in patients whose cancer biology is driven by the specific protein the drug targets. A notable historical example is the targeting of estrogen receptors and HER2 in breast cancer patients.

There has been a growing interest in expanding our understanding of cancer-related processes through advanced genetic analysis techniques. One such approach involves using comprehensive methods like whole exome sequencing (WES) or whole genome sequencing (WGS) to study the DNA, along with RNA sequencing (RNA Seq) to analyze the RNA molecules.

In the Oxford metanalysis, endocrine therapy by tamoxifen, an estrogen receptor modulator, decreased breast cancer mortality by around one-third in patients with early-stage breast cancer expressing estrogen receptors. No benefit was observed in patients without expression.^4^

Since this historical example, genomics has emerged as a tool to decipher which oncogenes drive cancer progression. Historical examples include HER2 amplifications in breast cancers, EGFR mutations in lung cancer, cKIT mutations in GIST, and BRAF mutations in patients with melanoma.^5,6^ In all these examples, a targeted therapy by HER2, EGFR, KIT, and BRAF signaling inhibitors improved the outcome of patients with metastatic cancer presenting the genomic alteration.

This allows optimal bioactivity with minimal toxicity. As example, drugs targeting specifically KRAS G12C mutations ^7^ and 542/545/1047 PIK3CA hotspot mutations ^8^ have been developed. The further evolution included the detection of more complex genomic alterations like gene fusions, and the detection of germline alterations driving cancer progression. Gene fusions have been described and used a companion diagnostic since very long time. As example PML-RAR fusions in acute myeloid leukemia defines a group of patients with outlier sensitivity to retinoic acid and arsenic. In the last few years, several gene fusions have been identified as driver alterations and represent now a biomarker for treatment decision. This includes FGFR2, NTRK, ALK, RET, ROS1 and NRG fusions.

While targeting oncogene is now an established strategy, the targeting of tumor suppressor gene (TSG) is a major challenge. Several approaches have been developed to address this topic. First, synthetic lethality has been a major success for BRCA-mutant cancers. Indeed, PARP inhibitors have been shown to improve outcome in patients with BRCA1/2 mutations and a breast, ovarian, prostate, pancreatic cancers.^5^ In the 2^nd^ approach, drugs have been used to inhibit the pathway activated by the loss of the tumor suppressor gene. As example, patients with PTEN mutations and metastatic breast cancer derive benefit from AKT inhibitors.^9^ Finally, in the last approach, investigators aim at reactivating the mutated TSG.

The example discussed above, showed that targeting a validated driver oncogene improves patient outcome. Nevertheless, this comes with several challenges.

### Resistance to targeted therapies

Firstly, treatment resistance inevitably occurs in most of the patients. Some co-mutations or protein expression leading to alternative signaling or feedback loops explain primary resistance to genomically driven targeted therapies. As example, KRAS mutations explained resistance to EGFR inhibitors in patients presenting a metastatic colon cancer.^10^ This led to identifying KRAS mutations as a biomarker of resistance performed in routine practice. At the opposite, EGFR expression has been reported to mediate resistance to BRAF inhibitors in patients with colon cancers, and targeting EGFR is now a mandatory treatment to optimize the use of BRAF inhibitors in patients with colon cancers.^11^

Examples abound, such as the utilization of MEK inhibitors alongside BRAF inhibitors in melanoma patients. Acquired resistance often stems from the emergence of new alterations conferring resistance to targeted therapies. The development of the EGFR T790M mutation following EGFR inhibitor treatment stands as a historical instance. Recent instances include the emergence of ESR1 mutations in breast cancer patients treated with endocrine therapy, AR fusions after anti-androgen treatment, or reverse BRCA mutations post-PARP inhibitor therapy.

The debate over whether these mutations originate from preexisting subclones or are induced by exposure to targeted therapy persists, and it is highly probable that both phenomena contribute to cancer development. Detecting preexisting resistant subclones prior to targeted therapy exposure could profoundly impact precision medicine by elucidating the eventual resistance mechanisms a tumor may develop. Drugs addressing these secondary mutations have shown clinically meaningful improvements in outcomes. Some of these compounds have now transitioned to the first-line setting with the aim of preemptively preventing secondary resistance while effectively targeting the initial driver mutation.

As an example, osimertinib, a third-generation EGFR TKI, was initially developed to target the frequent EGFR T790M resistance mutations and is now given to treatment-naïve patients with EGFR-activating mutations in the first-line setting.^12^ The next challenge in this field will be to block the mutational process leading to the subsequent type of resistance.

### Translating biomarker testing in clinical setting

Systematic and routine testing of these genomic alterations in daily practice represent the second challenge. Genome sequencing is the gold standard for assessing the previously mentioned genomic alterations, and the fastest and cheapest way of doing it is to perform multigene sequencing that assesses all alterations in a single assay. The clinical utility of multigene sequencing has been validated and this technology is now recommended in most of the cancer types. Several recent trials have suggested that the interpretation of genomic testing should be driven by dedicated frameworks, like ESCAT (ESMO Scale for Clinical Actionability of Molecular Targets) or ONcoKb, and that only validated genomic alterations should be considered for daily practice.^5^ The availability and use of biomolecular technologies in routine clinical practice remains heterogeneous and multifactorial, both within and across countries. Economic issues, barriers to access, and health system disparities limit their systematic interpretations. A recent ESMO (European Society of Medical Oncology) survey provides a comprehensive overview of the availability and accessibility of biomolecular technologies to patients in countries in the World Health Organization (WHO) European Region, formally demonstrating that comprehensive NGS panels remain largely inaccessible in clinical routine practice and are limited to clinical and translational research, even in EU and US area.

### *Improving* the genomic-driven targeted therapies development flow

The third challenge is to change the frameworks associated with the development of genomic-driven targeted therapies. While these drugs target genomic alterations and sometimes present outstanding efficacy, they are still developed using historical randomized trials in diseases defined by organ of origin (breast, colon…). Developing drugs using single-arm phase II trials and across tumor types could dramatically speed up drug development and access to therapies. For example, TRK inhibitors were shown to be effective in single-arm phase II trials that were agnostic for organ-of-origin.^13^ Additionally, comparing the evidence generated by single arm performed in rare disease entities, and in more frequent diseases in case of a very high magnitude of efficacy to synthetic matched real-world evidence arms would allow for a faster and broader registration of precision-oncology strategies, improving access to medication and care of all affected patients.

### Understanding cancer heterogeneity

To improve biomarker-driven therapies, the final challenge is enhancing genome sequencing with a broader biological assessment to boost accuracy and address tumor heterogeneity, with the characterization of co-mutations. Several technologies are being developed to address these needs. Spatial biology and organoid-based ex-vivo models represent cutting-edge technologies in medical research, reshaping diagnostics and predictive disease modeling. Aligned with genomics, these tools unravel unique biological landscapes, identifying therapeutic targets and deciphering molecular mechanisms in cancer progression.

For example, spatial insights derived from advanced technologies like spatial biology and organoid-based ex-vivo models play a pivotal role in refining drug development for biomarker-driven therapies. By combining genome sequencing with a comprehensive biological assessment, these insights contribute to increased accuracy and the resolution of tumor heterogeneity, particularly in characterizing co-mutations. These cutting-edge tools, aligned with genomics, unravel distinctive biological landscapes, aiding in the identification of precise therapeutic targets and the deciphering of molecular mechanisms in cancer progression. This spatial information enables the development of drugs with a more tailored treatment strategy, optimizing their effectiveness in addressing the complexities of individual cases.

Integrating assessments into multidimensional scores promises a nuanced understanding of disease biology within individual patients over time, heralding a new era in precision medicine.^14^

Results of the assessments of these new dimensions of biology will be integrated in multidimensional scores that will ultimately recapitulate the biology of the disease in a defined patient over time.

In summary, the evolution of cancer research from oncogenes to advanced genetic analysis techniques like whole exome sequencing (WES), whole genome sequencing (WGS), and RNA sequencing (RNA Seq) has illuminated the intricacies of molecular pathways, facilitating targeted therapies. Despite successes, challenges persist. Identifying predictive biomarkers for resistance, implementing routine genomic testing, redefining drug development frameworks, and embracing a multidimensional approach to complement genomics are critical frontiers. Bridging these gaps holds the promise of accelerating drug development, enhancing treatment precision, and comprehensively understanding cancer biology, ultimately reshaping the landscape of precision medicine.

## The Evaluation of Molecular Techniques for Biomarker Analysis

Clinical biomarker testing relies on detection of molecules such as DNA, RNA, protein metabolites or transcription factors that are either produced by the tumor or as a response to the tumor that can help with diagnosis, as well as provide predictive and prognostic information to guide patient care. The evolution of molecular techniques with efforts to develop sensitive and specific biomarkers using novel analytical testing methods have contributed to the growing field of precision oncology. Some of the commonly used testing methods in clinical diagnostic practice are described below and shown in Table 1.

Immunohistochemistry (IHC) is a widely available, rapid, and relatively inexpensive testing methodology for detecting proteins expressed by specific cells in tissue specimens. While IHC has been used since the 1940s to visualize antigen-antibody interactions, frequently conjugated to an enzyme such as peroxidase (chromogenic IHC), other techniques such as tagging to a fluorophore (immunofluorescence), multiplex IHC with different antibodies used to stain the same tissue section, multiplex immunofluorescence (mIF), and newer technologies such as tyramide signal amplification and fluorescent quantum dot nanocrystals, with higher sensitivity can be employed to detect and quantify specific protein expression.^15–18^ For example, IHC is the recommended assay for assessment of estrogen receptor (ER) in breast cancer, the first established predictive biomarker, and the most widely used predictive marker for consideration of endocrine therapy in breast cancer patients. Several other predictive/prognostic IHC-based biomarkers such as progesterone receptor (PR), human epidermal growth factor receptor 2 (HER2), Ki-67, anaplastic lymphoma kinase (ALK), Ros proto-oncogene tyrosine-protein kinase (ROS1), DNA mismatch repair protein (MMR), Neurotrophic tyrosine receptor kinase (NTRK), and programmed death-ligand 1 (PD-L1) are routinely used in clinical practice for a variety of solid tumors.

Enzyme-linked immunosorbent assay (ELISA) is a commonly used immunological assay for detecting and measuring antibodies, antigens, and proteins in clinical practice, especially in blood and other body fluids. Newer techniques, such as electrochemical ELISA, have increased sensitivity for detecting low-abundance protein biomarkers.^19,20^ Some commonly used ELISA assays for detecting and estimating tumor marker levels include prostate-specific antigen and carcinoembryonic antigen (CEA) in patients with prostate cancer and pancreatic/colon cancer, respectively.

Fluorescence *in situ* hybridization (FISH) is a routinely used technique that uses fluorescently labeled probes that can hybridize with a nucleic acid sequence to detect gene copy number changes (for instance, amplification) or gene rearrangements/fusions in tumor cells. Additional use of FISH techniques to detect diagnostic, predictive, and prognostic biomarkers include multiplex FISH and comparative genomic hybridization.^21^ One of the most widely used FISH-based biomarker assays is evaluating HER2 amplification. HER2 is overexpressed in up to 20-30% of breast cancer patients as well in several other malignancies including gastric and gastro-esophageal cancer, colorectal cancer, ovarian cancer, prostate cancer, and lung cancer. ^22–24^ While HER2 can be reliably assessed using IHC, indeterminate staining with IHC (2+) is typically reflexed to FISH. Other predictive/prognostic FISH-based biomarkers that are frequently used in clinical practice include *ALK, ROS1, NTRK*, rearranged during transfection (*RET*) and MET proto-oncogene, receptor tyrosine kinase (*MET*).

PCR-based genomic profiling is the most common molecular methodology used for both DNA- and RNA-based applications in oncology. PCR-based amplification in combination with sequencing has been widely used for the detection of DNA mutations, gene fusions, copy number alterations, DNA methylation analysis in a variety of tumor types. Various modifications, including real-time PCR and digital PCR have been employed in clinical practice to increase the sensitivity of detecting biomarkers.

Next-generation sequencing (NGS) combines unique sequencing chemistries with bioinformatics, to allow for massively parallel sequencing with high throughput and scalability. NGS is routinely used for detecting both germline variants and somatic mutations for DNA-based testing, as well as RNA-based biomarkers, such as gene fusions and RNA sequencing.^25–27^ NGS can be performed using either an amplicon-based method with primer panels that amplify gene fragments harboring driver mutations, or targeted capture and hybridization of regions of interest for sequencing using capture probes. The last couple of decades have witnessed an increasing need for an expanded range of biomarker tests that are needed to tailor therapy based on specific molecular and immune characteristics of the tumor. Therefore, in the face of a growing need for more complex biomarker information, comprehensive genomic profiling platforms such as NGS-based assays are largely being preferred over single-gene testing.

Several gene expression profiling assays are currently being used to evaluate gene expression and transcriptome changes for classifying tumors into molecular subtypes that can be used for predictive or prognostic purposes. Gene expression microarrays are commonly used to evaluate differentially expressed genes in tumor samples and are used to assess prognosis and recurrence of disease and can serve as predictive biomarkers of therapeutic response. Microarray-based molecular classifications of breast cancer patients are used to predict recurrence risk and chemotherapy response.^28,29^

Advancements in molecular techniques drive precision oncology’s biomarker analysis. Immunohistochemistry, enzyme-linked immunosorbent assay, fluorescence in situ hybridization, polymerase chain reaction, and next-generation sequencing are vital tools, each playing a unique role. From protein detection to comprehensive genomic profiling, these techniques contribute to the evolving landscape of precision medicine, offering insights for tailored therapeutic approaches and enhancing our understanding of tumor biology.

## Biomarkers to Characterize Immune Response

Lung cancer serves as a key example for development of biomarkers to characterize immune responses in cancer therapy. Immunotherapy has gained prominence in the treatment of non-small cell lung cancer (NSCLC), encompassing early-stage, locally advanced, and metastatic cases.^30^ Despite the progression free and overall survival benefits derived from the use of checkpoint inhibition, the number of patients benefitting from durable disease-control is limited.^30,31^ Therefore, stratification using biomarkers is important to avoid over- and under-treatment of patients. Commonly utilized biomarkers include PD-L1 status^32^, tumour mutation burden (TMB)^33^ and more recently, pathological response after neoadjuvant chemoimmunotherapy^34^. However, each of these biomarkers has limitations, and as our understanding of cancer-immune interactions evolves, new potential biomarkers are emerging. These biomarkers can be broadly categorised as tumour cell intrinsic, or microenvironment related. The optimal integration of these markers, especially in conjunction with emerging immunotherapies like vaccines, T-cell receptor engagers, and cellular therapies, remains to be determined.

### Tumour cell intrinsic features

The immune system can recognize diverse tumour antigens, including somatic mutation-derived neoantigens, oncoviral antigens (e.g., HPV E6), lineage-related proteins (e.g., Melan-A in melanoma), and cancer-testis antigens.

TMB reflects the potential neoantigen repertoire size, which has been shown to be targeted by checkpoint inhibitor-induced T-cell responses.^35,36^ It is estimated that ≤1-5% somatic mutations generate immunogenic neoantigens.^37–42^ Thus, a higher TMB increases the likelihood of generating immunogenic neoantigens and a productive T-cell response^43–48^. Cancers with a high mutation load^49^ such as NSCLC, head and neck cancers, melanomas, urothelial cancers, and microsatellite unstable tumours often respond favourably to immunotherapy. TMB assessment is prognostic for response to immunotherapy and the FDA has approved the use of pembrolizumab for high TMB solid cancers (defined as ≥10 mutations/megabase using the FoundationOne CDx assay)^50^. A major challenge in using TMB as a biomarker is how to define the cut-off for TMB-high tumors with varying panel designs and bioinformatics pipelines. Whilst the feasibility of blood based TMB measurements has been demonstrated^51–53^, these assays cannot be used in non-ctDNA (circulating tumor DNA) shedding disease and may not be accurate in low ctDNA shedding disease^54^. Efforts to standardize TMB calculation in clinical assays are ongoing^54,55^.

Recent developments have highlighted that not all somatic mutations are equal. Cancers continue to acquire somatic mutations through their lifetime, with some being ‘clonal’ (present in every cell) and others ‘subclonal’. Clonal neoantigen burden correlated with durable responses in NSCLC treated with checkpoint inhibitors^56^. Furthermore, clonal TMB was the biomarker with the most substantial effect size in predicting radiological tumour response across 1008 checkpoint-inhibitor treated cancers (CPI1000 cohort) comprising of NSCLC, melanoma, renal, urothelial, breast, colorectal and head and neck cancers^57^. By contrast, subclonal TMB had no significant association with tumor response to immunotherapy drugs (e.g. immune checkpoint inhibitor), suggesting the clonal portion of TMB may be critical in immunotherapeutic response.

The type of mutation and the etiology driving mutagenesis are also important for responses to immune checkpoint inhibitor. Insertions/deletions (INDELs), particularly frameshift variants, can generate 3x more high affinity neoantigens per variant compared to a non-synonymous mutation^58^. Both INDEL TMB, and nonsense-mediated decay escaping frameshift INDEL burden correlate with checkpoint inhibitor responses^57^. The presence of a smoking mutational signature was also associated with checkpoint response in the lung subset of the CPI1000 cohort, independent of TMB burden^57^. This might reflect the dinucleotide mutation burden associated with smoking, increasing the likelihood of mutating two amino acids and/or causing more radical amino acid substitutions^57^ for the immune system to detect.

Another source of antigens of interest in NSCLC are cancer/testis antigens (CTA), such as MAGE, PRAME, or NY-ESO-1^59^. They are immunogenic ^60,61^, frequently expressed in NSCLC^62,63^, and their expression in normal tissues is restricted to immune-privileged sites that harbour germ cells. Off the shelf vaccine^64–67^ and cellular therapies^68,69^ have been developed to target these in multiple cancer types, and detecting these antigens is usually a pre-requisite biomarker for recruitment to such clinical studies.

### Genomic background of the cancer

In addition to serving as potential neoantigens, some somatic alterations may confer differing sensitivities to immune checkpoint inhibition. For example, *EGFR*- and *ALK*-driven lung cancers are associated with resistance to checkpoint inhibitors^70^. Whilst this may partially relate to a reduced TMB^71^ due to enrichment in non-smoker patients, a non-inflamed tumour microenvironment with reduced tumour infiltrating lymphocytes^72^ and reduced interferon gamma (IFNγ) signature may also contribute to this insensitivity^73^, and also to the activation of CD73 adenosine pathway, as a potential therapeutic target for EGFR-mutant NSCLC.^74^

The literature on PD-L1 expression in *EGFR*-mutant cancers is conflicting^71,73,75–79^, though pooled analyses suggest that PD-L1 expression might be reduced compared to *EGFR*-wild type cancers^71,75^. Conversely, *MET* exon 14 skipping mutations are associated with high PD-L1 expression^79,80^, and co-mutated *TP53*/*KRAS* NSCLCs are associated with increased sensitivity to checkpoint blockade^81^. However, if the *KRAS*-mutant tumor also harboured an *STK11* mutation, this was associated with reduced PD-L1 levels; or if it harboured a *KEAP* mutation, there was an associated reduction in T-cell infiltration and downregulation of inflammatory cytokines^70,79,82,83^. Both these variants confer worse clinical outcomes with checkpoint inhibition^82^. Similarly, loss of *PTEN* is associated with increased expression of immunosuppressive chemokines, decreased T-cell infiltration, reduced IFNγ expression, and worse outcomes with PD-1 targeted therapy in melanoma^84^ and NSCLC^85^. Mutations in DNA damage repair pathways such as *POLE, POLD1* and *MSH2* are also associated with response with checkpoint inhibitors, likely reflecting increased TMB^61^. Mutations in BRCA1/2^86,87^ and other genes within the homologous repair pathways^87^, as well as mutations in MUTYH^69^ are associated with increased TMB and tumour-infiltrating lymphocytes. Pan-cancer analyses also demonstrated that copy number gains and losses of certain genes may be associated with altered sensitivity to checkpoint blockade; for example, *CCND1* amplification was associated with resistance, whereas 9q34.3 loss, encompassing *TRAF2*, was associated with increased sensitivity^56^. These examples demonstrate the potential utility of the tumour’s genomic background as a biomarker for checkpoint blockade response.

### Other Mechanisms of Immune Evasion

Tumour cells employ various immune evasion strategies, impacting immunotherapy responsiveness. These include increased PD-L1 expression, alteration in antigen presentation machinery (e.g. *B2M* or loss of heterozygosity in human leukocyte antigen (HLA LOH)), and loss of IFNγ sensitivity.

PD-L1 expression serves as a clinically validated biomarker for immunotherapy in NSCLC^32^, but not SCLC^89^. There are three FDA approved PD-L1 immunohistochemistry assays currently used as companion diagnostics: Dako 28-8 for the use of ipilimumab/nivolumab in NSCLC; Dako 22C3 for the use of pembrolizumab with multiple solid tumours; and Ventana SP142 for the use of atezolizumab with NSCLC, urothelial malignancies and triple-negative breast cancer^32^. In advanced NSCLC, and in the absence of driver *EGFR* and *ALK* variants, a PD-L1 ≥1% cut-off is currently used to determine suitability for combination nivolumab/ipilimumab^71^ or pembrolizumab monotherapy^13,72^; and a PD-L1 ≥50% cut-off is used to determine suitability for atezolizumab monotherapy^51^ or cemiplimab monotherapy^92^. Interestingly, high PD-L1 expression does not appear to be significantly associated with high TMB, suggesting that a combination of these two biomarkers may better than using either one alone^93^.

Aberrations in the antigen processing and presenting pathway are associated with poorer responses to checkpoint blockade^94^. An ‘antigen processing machine’ score generated from expression data of genes including *B2M, CALR, NLRC5, PSMB9, PSME1, PSME3, RFX5*, and *HSP90AB1* was associated with response to checkpoint blockade in NSCLC^94^. Mutations or loss of *B2M*, encoding ß_2_-microglobulin, an extracellular component of the major histocompatibility complex class I (MHC I) molecule which stabilises cell surface expression of MHC I and plays a role in presenting antigenic peptides to the immune system^95^, have been implicated in acquired resistance to immunotherapy in melanoma^96,97^ and NSCLC^98^. Therefore, identifying these changes in *B2M* may provide an important biomarker for response to checkpoint blockade. Another strategy for cancer cells to reduce neoantigen expression would be through the loss of genomic segments harbouring immunogenic mutations, or through genomic loss or repression of HLA allele expression^99,100^. Work on NSCLC tumours demonstrated that *B2M* mutations tend to be mutually exclusive with HLA LOH and alterations in other components of antigen presentation machinery^80^. The presence of HLA LOH is enriched in ‘immune hot’ tumours, suggesting that it is a mechanism of immune evasion^99^. Thus, understanding the expression of HLA alleles would be particularly important when designing personalised neoantigen-targeting treatments such as vaccines and cellular-based therapies.

*JAK1* and *JAK2* genes encode signal transducers that respond to IFNγ by increasing antigen presentation, producing chemokines to attract T-cells, and triggering tumour cell apoptosis^101,102^. *JAK1/2* signalling also results in increased PD-L1 expression thereby allowing escape from T-cell mediated cytotoxicity^103^. Mutations or loss in *JAK1/2* would therefore result in reduced response to IFNγ signalling, increased tumour growth, reduced T-cell infiltration and futility in using PD-1/PD-L1 targeting agents^104^. Indeed, *JAK1/2* mutations are associated with primary resistance to checkpoint blockade in melanoma ^97, 104–106^.

### Microenvironmental features

The tumour microenvironment comprises immune and stromal cells. The roles various immune cells, such as tumour infiltrating lymphocytes (TILs), regulatory T-cells, B-cells, neutrophils, eosinophils, macrophages and dendritic cells, in promoting or suppressing tumour growth are subject to ongoing research^107,108^. The presence of these cells and their associated chemokine secretory signatures are being developed into potential biomarkers for immunotherapy responsiveness.

One of the most studied microenvironment-related biomarkers in NSCLC is TIL infiltration, with results suggesting that high infiltration, particularly those of CD8+ cells, is associated with better overall outcomes^109,110^, and increased sensitivity to checkpoint blockade^100–119^. TILs have demonstrated a more robust correlation with the response to checkpoint blockade compared to TMB levels in patients with PD-L1–negative disease^115^. The investigation into whether enhancing TIL assessment through staining for co-expressed markers like PD-1 or CD39 can optimize the predictive performance of these biomarkers for immunotherapy drug responses.^120,121^

Nevertheless, TILs can be assessed by pathologists through straightforward hematoxylin and eosin staining (H&E) slides, facilitating seamless integration into clinical practice, akin to its established use in breast cancer^103^.

Conversely, other cells, such as T-regulatory cells, M2 macrophages, and myeloid derived suppressor cells (e.g., neutrophils) are associated with immunosuppressive environments ^107,108,123–130^. Circulating markers such as high baseline neutrophil-to-lymphocyte ratio or low eosinophil counts seem to correlate with worse outcomes with checkpoint blockade^131–138^, and can also be easily incorporated into clinical practice.

To account for the multiple cell types within the immune microenvironment, as well as their activation and chemokine signaling, multiple transcriptomic signatures have been developed to predict response to checkpoint blockade. These vary in the platforms used, the numbers of genes assessed and the pathways involved: from T-cell activation and cytolytic activity, to IFNγ signalling, to antigen processing and B-cell responses^139–146^. Chemokines incorporated into these panels include *CXCL9, CXCL10, CXCL11, CXCL13* and IFNγ. Pan-cancer analyses in the CPI1000 cohort identified *CXCL13* as being highly and differentially expressed in responders to checkpoint blockade compared to non-responders, and *CXCL9* having the largest effect size for association with response when compared to other markers of tumour infiltration^56^.

### Host factors

Host-related factors also play a role in sensitivity to checkpoint blockade. For example, biological male sex appears to confer benefit when considering checkpoint blockade^147^. Several germline genetic variations appear to contribute to immune traits^148,149^. It is estimated that 15-20% of intratumour variability in T cell infiltration and IFNγ signalling is heritable. For example, polymorphisms in *RBL1* are associated with T cell infiltration, and those in *IFIH1, STING1*, and *TMEM108* seem to affect IFNγ signalling^150^. Polymorphisms in *IFT74, DCDC2* and *NRSN1* are also associated with altered CD8+ T cell phenotypes^151^ and those in *CTLA4, CCR5, IRF5, CTSS* seem to impact immunotherapy response^152^.

The germline HLA status of patients may also impact response to checkpoint blockade. Heterozygosity at all loci of *HLA-A, B* and *C* was associated with better survival outcomes than homozygosity in one of the alleles, likely reflecting the ability of the immune system to recognise a wider repertoire of antigens^153.154^.

In patients with different variants of the HLA genes (heterozygous HLA alleles), the success of checkpoint blockade immunotherapy is linked to the evolutionary divergence of these genetic variants, measured by something called the Grantham distance. Essentially, the Grantham distance helps quantify how much these genetic variants have evolved over time. Surprisingly, individuals with higher evolutionary divergence in their HLA alleles had better responses to checkpoint blockade treatments compared to those with less divergent alleles. This discovery underscores the importance of understanding the evolutionary aspects of our immune system’s genetic makeup in predicting the effectiveness of immunotherapy, shedding light on a potentially significant factor influencing patient outcomes. ^153,154^

This was seen in NSCLC and melanoma. Furthermore, specific alleles were associated with response to anti-CTLA4 treatments in melanoma, with HLA-B44 supertype being associated with better prognosis and HLA-B62 supertype being associated with poor outcome^154^. These results suggest that germline genotype can help predict response to immunotherapy and is an important consideration in neoantigen based therapeutic approaches. Indeed, neoantigen prediction pipelines rely on genotyping the HLA alleles^155,156^.

In this specific historical period of cancer treatment, characterized by the increasing use of immunotherapy drugs, understanding the immune response has become a daily challenge that, originating from research, has seamlessly integrated into daily clinical practice. Identifying and evaluating biomarkers, including PD-L1 status, tumor mutational burden (TMB), and other factors related to the microenvironment and genomic background, are crucial for shaping personalized treatment strategies and effectively stratifying patients. This comprehensive approach is essential for maximizing therapeutic outcomes in the era of immunotherapy. Furthermore, this understanding pave the way for advancements in cancer diagnostics, encompassing both tissue and liquid biopsy strategies, ultimately improving the precision and efficacy of diagnostic approaches in the evolving landscape of cancer care.

## Current Perspective of Blood-Based Biomarkers

In recent years, the study of blood-based biomarkers has advanced dramatically, especially in the area of circulating tumor DNA (ctDNA). Based on a large body of work, it is now clear that ctDNA most often comprises a variable, although small, fraction of the total circulating cell-free DNA (cfDNA) in the plasma of cancer patients. This fraction is correlated with a variety of biological factors including tumor type, histology, disease burden, cell proliferation, and genomic instability^158–162^. The main clinical applications of ctDNA analysis include noninvasive tumor genotyping, monitoring response to therapy, detection of minimal residual disease (MRD) following treatment, and early cancer detection. Each of these applications targets a unique patient population with a distinct distribution of ctDNA concentrations, requiring careful consideration of the required limit of detection (LOD) for assays. Patients with advanced disease have the highest average ctDNA levels (often >1%), allowing tumor variant identification directly from the plasma with assays that have relatively less sensitive LODs. However, in the setting of MRD detection or screening for early-stage tumors, ctDNA levels can be below one part per million, necessitating much more sensitive assays.

The first ctDNA application that became part of the standard of care was non-invasive tumor genotyping to identify therapeutically actionable mutations in patients with advanced disease. Increasingly, liquid biopsies are performed in parallel with tumor tissue biopsies due to the combination identifying more actionable mutations and liquid biopsies having faster turnaround times^163,164^. One key advantage of liquid over tissue biopsies is that ctDNA can contain contributions from multiple tumor deposits and may therefore better capture tumor heterogeneity than a tissue sample from a single site. Liquid biopsies can enable tumor genotyping in patients for whom a tissue biopsy is not available, or when the biopsy sample contains low tumor cellularity.

Initially implemented using quantitative and digital PCR assays^165–168^, ctDNA genotyping is now predominantly performed via next-generation sequencing (NGS) panels that interrogate an array of clinically actionable mutations. The limit of detection of ctDNA genotyping assays for individual mutations is ~0.2%^169,170^, making them suitable for application to patients with metastatic disease who generally have pre-treatment ctDNA concentrations of ~1-5%^158^. Studies in multiple cancer types have demonstrated high concordance between ctDNA and tissue genotyping^171–175^ and plasma-based genotyping has been found to recapitulate the expected tumor mutational landscape in large, multi-center cohorts^176^. In addition, prospective studies have demonstrated that the addition of pre-treatment ctDNA analysis to tissue-based genotyping can increase the detection of therapeutically actionable mutations in lung^163^,^177^, breast^178^, and colorectal^179^ cancers. Therefore, performing liquid biopsy-based genotyping first or in parallel with tissue-based genotyping has been recommended in certain advanced disease settings by multiple expert groups^180,181^. Importantly, false-negative results due to low ctDNA abundance represent a key limitation of plasma-based genotyping, and reflex tissue testing is therefore strongly recommended in parallel or when no tumor mutations are found using a liquid biopsy first approach, imposing some delays.

Genotyping via ctDNA can also be used at the time of progression in order to identify resistance mechanisms that might be therapeutically actionable^180^. For example, in patients with EGFR-mutant NSCLC treated with tyrosine kinase inhibitors, ctDNA analysis at the time of resistance can identify mechanisms of resistance such as secondary EGFR mutations and MET amplification^182,184^. Separately, tumor mutational burden (TMB) and microsatellite instability (MSI), which are potential predictors of immunotherapy response, can be identified using ctDNA analysis in patients with advanced disease. Blood-based TMB (bTMB) measurements correlate with those determined from tumor tissue ^183,185^ and higher bTMB was associated with better response to atezolizumab in advanced NSCLC^186^.

Beyond genotyping applications, another potential use of ctDNA in patients with metastatic disease is monitoring ctDNA dynamics over the course of treatment. Measuring ctDNA concentrations during treatment as a proxy for total tumor burden can be used to assess therapeutic efficacy. In many disease and treatment contexts, a drop in detectable ctDNA during treatment has been associated with clinical benefit^187,193^. This approach may allow identifying non-responders who might benefit from escalation of therapy earlier than the radiological follow-up, and trials testing such strategies are underway^194^. This strategy remains to be formally established in this palliative context though.

In patients with early-stage disease, two promising applications of ctDNA analysis are detection of MRD and cancer screening. In both scenarios, ctDNA levels are significantly lower than in patients with metastatic disease. The median pre-treatment ctDNA concentration in stage I lung adenocarcinomas, for example, is approximately 1 part per million^161,162,195^. Similarly, in localized lung cancer patients treated with curative intent, ctDNA MRD concentrations can be 1 part per million or lower^195,196^. These exquisitely low ctDNA levels necessitate assays with a much more sensitive LODs than are attainable by ctDNA-based genotyping assays.

The LOD of ctDNA MRD assays can be improved through several strategies, including leveraging prior knowledge of tumor mutations from previous tissue or plasma sequencing (i.e. tumor-informed ctDNA analysis), tracking multiple mutations, increasing the amount of plasma input, and reducing errors resulting from technical artifacts introduced during library preparation and sequencing^197^. The most sensitive MRD methods employ a personalized, tumor-informed approach, in which a pre-treatment tumor sample is first sequenced to identify patient-specific mutations that are then tracked in the plasma over the course of treatment. These assays have limits of detection of ~0.01%, approximately 100 times more sensitive than the plasma genotyping assays discussed above. ctDNA MRD measured using tumor-informed assays at a landmark timepoint in the weeks following curative-intent treatment has been shown to be a powerful and early indicator of prognosis in early-stage lung^162,198^ breast^199–201^, and colorectal cancers^191,202–204^, among others^197^, usually with a significant lead time over imaging. Across studies and tumor types, patients with undetectable ctDNA after treatment have dramatically better outcomes than those who remain ctDNA-positive.

Importantly, while the positive predictive value of tumor-informed first generation ctDNA MRD assays is high, their sensitivity is suboptimal. For example, first generation assays can only detect MRD at the post-treatment landmark in approximately one third of early-stage NSCLC and half of colon cancer patients destined to develop recurrence ^159,162,197,204–206^. This sensitivity issue implies that while first generation ctDNA MRD assays could potentially be used to escalate adjuvant therapy in MRD-positive patients, withholding standard of care adjuvant treatment in MRD-negative patients would result in undertreating a large fraction of patients who could benefit. Therefore, there is great interest in developing even more sensitive MRD assays, such as the recently described PhasED-Seq method which tracks phased somatic mutations and has an LOD below one part per million^195,196^. Future studies will strictly be required to test if these more sensitive approaches could be used to safely de-escalate treatment in MRD-negative patients, in a curative setting.

Early detection of cancers in asymptomatic individuals using approved screening modalities can decrease cancer-specific mortality and therefore there has been intense interest in recent years in developing liquid biopsy-based screening tests. However, as mentioned above, ctDNA levels in early-stage cancer patients are usually extremely low, often below one part per million. However, unlike MRD analysis, where prior knowledge of tumor mutations can be used to increase assay sensitivity, screening methods must employ tumor-naive approaches. For this reason, existing ctDNA early detection assays cannot attain the same LODs as the tumor-informed MRD assays and instead have LODs more similar to ctDNA genotyping assays (~0.1%). Furthermore, screening assays require high specificity, since the majority of individuals being screened will not have cancer and therefore low specificity would result in the majority of positive tests being false positives.

Even in light of these challenges, numerous commercial and academic groups have demonstrated the potential of ctDNA analysis to detect early-stage cancers^161,207–210^. These studies have employed a variety of technical approaches, including analysis of somatic mutations, cfDNA methylation, cfDNA fragmentation patterns, and combinations of multiple approaches. While promising and in very fast development, ctDNA-based early detection methods continue to have important shortcomings. One recurring issue is that performance of assays is often overly optimistic in the initial studies that report them and degrades in subsequent validation studies. Additionally, given the relatively poor LOD that can currently be achieved by tumor-naive approaches, it is not surprising that sensitivity for early-stage cancer remains suboptimal. For example, the Grail Galleri test, which is based on DNA methylation and is clinically available, has a sensitivity of only 7% for stage I lung adenocarcinoma^211^. Therefore, there remains a major unmet need for further technical advances that improve the sensitivity of tumor-naïve ctDNA assays. Ultimately, large, randomized studies will be required to determine whether or not liquid biopsy-based early detection can achieve cancer-specific mortality benefits.

Although much cancer biomarker work in recent years has focused on ctDNA, detection of tumor-derived circulating RNA and novel protein-based technologies are two additional approaches that hold promise for developing clinically useful biomarkers. Though early cell-free RNA (cfRNA) studies primarily focused on small microRNAs as potential cancer biomarkers^212,213^, more recently several studies have reported proof of concept data demonstrating that messenger RNA (mRNA) can also be detected in plasma of healthy controls and cancer patients^59–61^. These studies have begun to reveal the cellular origins of cfRNA, finding that while the majority of cfRNA derives from hematopoietic cells, solid tissues also contribute cfRNA to the plasma^214–216^. A recent study that compared cfRNA in patients with lung or breast cancer to healthy controls found that plasma transcriptomes reflect the tumor’s tissue-of-origin^215^, suggesting that cfRNA may have utility in the detection, diagnosis, and monitoring of malignancies.

Plasma proteins have a long history as cancer biomarkers and are used routinely in the clinical setting. However, application of next generation protein assays has lagged behind next generation sequencing of nucleic acids for liquid biopsy assay development. Though mass spectrometry remains the primary tool for analyzing the proteome, it suffers from suboptimal sensitivity for low-abundance proteins, which usually include the key cancer-associated proteins to be measured. Recent breakthroughs in targeted multiplexed protein detection assays have facilitated the query of thousands of proteins with improved sensitivity for low-abundance proteins^217^. Two in particular that show promise for liquid biopsy applications are the proximity extension assay (PEA)^218^ from Olink Proteomics and the slow off-rate modified aptamer assay (SOMAscan) from SOMAlogic^219^. Using proximity ligation or DNA-based aptamers to capture protein targets of interest, respectively, both assays can measure thousands of proteins of varying abundance from a small biofluid sample. Recent studies have demonstrated the ability of these techniques to uncover cancer-related proteomic signals^220–222^. Compared to ctDNA, liquid biopsy proteomics and transcriptomics are early in their development, but recent advances suggest that these analytes may also play a more prominent role in the field in the future.

One of the crucial objective of cancer research has perpetually been the utilization of liquid biopsy in daily clinical practice. Particularly, the ctDNA analysis has transformed cancer diagnostics. Liquid biopsies, conducted alongside traditional tissue biopsies, offer advantages such as capturing tumor heterogeneity, monitoring treatment response, detecting minimal residual disease (MRD), and early cancer detection. Beyond ctDNA, emerging approaches involving circulating RNA and advanced protein detection hold potential, requiring further innovation and large-scale studies to determine clinical utility and impact on cancer-specific mortality.

## Conclusions

In the rapidly evolving field of cancer therapeutics, biomarkers are increasingly recognized as central to advancing precision medicine, signifying a transformative phase in oncology. This manuscript highlights the diagnostic capabilities of biomarkers, such as circulating tumor DNA (ctDNA) and immune profiling, as integral to personalized cancer treatment strategies. Biomarkers, especially ctDNA, are set to revolutionize cancer therapy by enabling precise identification of genetic mutations, thereby allowing for treatment personalization that enhances efficacy and minimizes side effects. The adoption of ctDNA in therapeutic protocols marks a shift towards a strategy where biomarkers not only aid in diagnosis but also direct therapeutic choices, monitor residual disease, and track cancer evolution and heterogeneity in response to treatment resistance over time.

The integration of immune-profiling biomarkers with cancer therapies introduces a nuanced layer to treatment modalities, leveraging the complex interaction between the immune system and therapeutic agents to improve treatment outcomes. This approach underscores the importance of precise patient selection for the effectiveness of new therapeutic strategies and the development of synergistic, more potent treatments.

Predictive biomarkers are emerging as essential tools in optimizing treatment strategies, ensuring that therapies are not only effective but also specifically tailored to the molecular profiles of individual patients, thereby maximizing benefits and minimizing risks. This is crucial for the progression of precision oncology.

Moreover, the narrative around biomarker-agnostic strategies in the context of Antibody-Drug Conjugates (ADCs) suggests a nuanced paradigm. Such strategies recognize that certain ADCs might not strictly require a specific biomarker for efficacy, broadening their applicability but also highlighting the need for further biomarker discovery to enhance their activity and accessibility.

The potential for integrating germline genomics as predictive biomarkers opens up new avenues for personalizing cancer treatment, further refining therapeutic decisions based on genetic insights. This evolution towards incorporating both germline and somatic biomarkers, such as ctDNA, signifies a strategic pivot in cancer treatment, enriching our understanding of cancer progression, heterogeneity, and treatment resistance.

In summary, the future of biomarkers in cancer therapeutics is brimming with promise, elevating their function from mere diagnostic tools to indispensable orchestrators of personalized treatment paradigms. Navigating this future demands a nuanced grasp of the complex dynamics between biomarker-based and biomarker-agnostic strategies, stressing the need for exceptionally specialized and rigorously applied oncological care. Simultaneously, it calls for an immediate and well-supported dialogue among political, academic, and industry circles to improve global access to molecular biomarkers. Through collaborative efforts among policymakers, researchers, and industry participants, we can mobilize resources to overcome access barriers and disparities. This holistic vision not only addresses the immediate requirements for efficacious cancer care but also lays the groundwork for sustained advancement in personalized treatments. Ultimately, this collective endeavor is poised to transform cancer therapeutics, ensuring positive outcomes for patients worldwide. This comprehensive vision paves the way for more effective and individualized treatments for all cancer patients, heralding a new era in the fight against cancer.

## Figures and Tables

**Figure:**
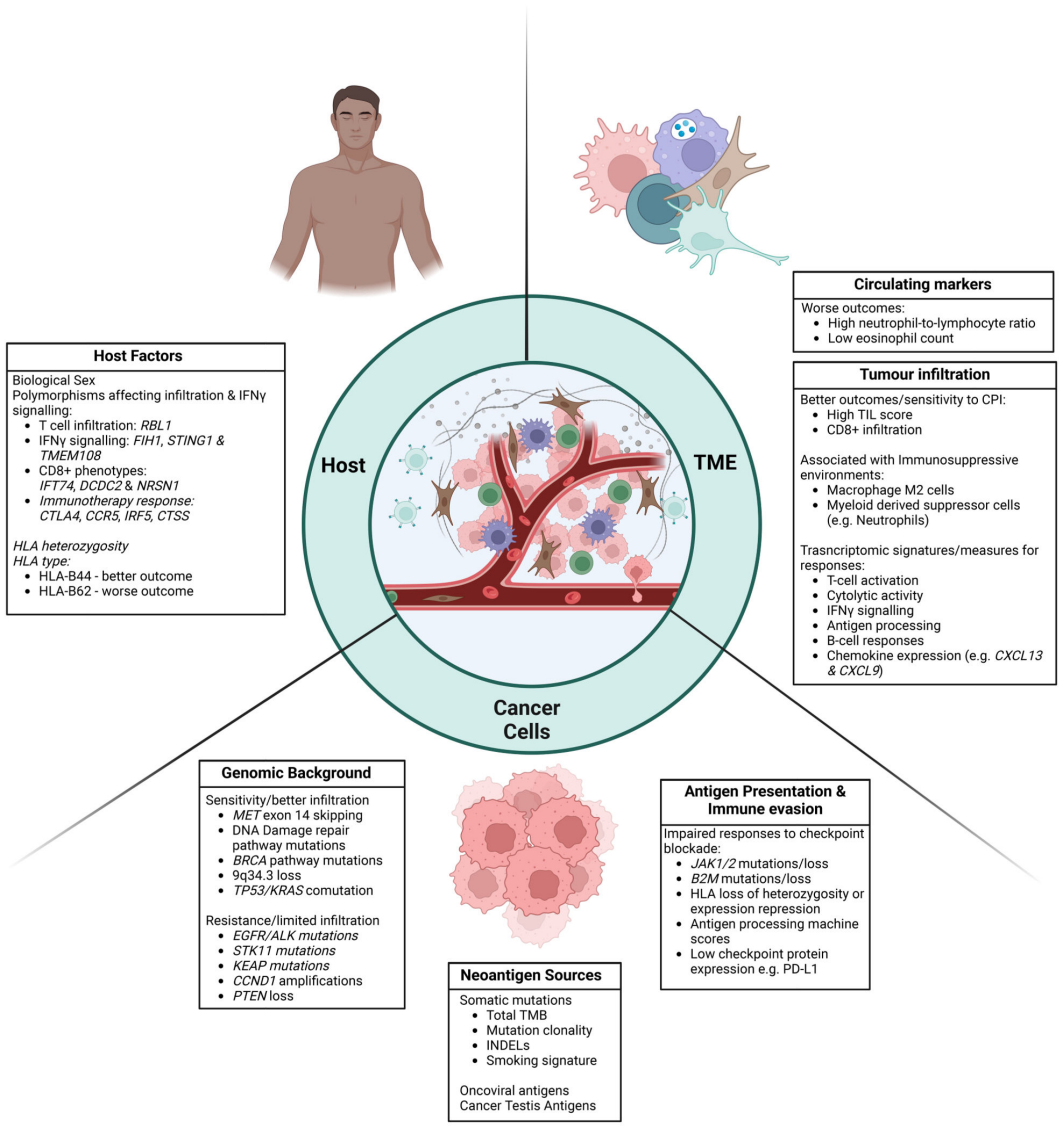


**Table 1:** Molecular Methods for Biomarker Testing in Solid Tumors

Technique	Application	Advantages	Limitations
IHC	Protein-based assay for detection of expression	Cheap, rapid, widely available Direct visualization of protein expression	Antibody availability Subjective interpretation/quantification
FISH	Hybridization using fluorescent-labeled probes to detect gene copy number changes or gene rearrangements/fusions	Relatively simple assay design Direct visualization of signals within cells of interest	Probe availability Restricted to specific locus/gene tested
PCR	Detection of targeted gene mutations, fusions, copy number alterations, DNA methylation	High sensitivity and specificity Relatively simple assay design Relatively low-cost	Limited throughput Restricted to targeted genes and regions of interest interrogated
NGS	Massively parallel sequencing of multiple genes for detecting mutations, fusions, copy number alterations	High throughput High sensitivity and specificity Comprehensive coverage Site/tumor-specific applications	High complexity Bioinformatics requirements Longer turnaround time
GEP	Differential gene expression between tumor/normal or pre/post-treated tumor	High throughput	Bioinformatics requirements Restricted to targeted genes

Abbreviations: FISH, fluorescence in situ hybridization; GEP, gene expression profiling; IHC, immunohistochemistry; NGS, next-generation sequencing; PCR, polymerase chain reaction
